# Assessing disease disclosure in adults with cystic fibrosis: the Adult Data for Understanding Lifestyle and Transitions (ADULT) survey Disclosure of disease in adults with cystic fibrosis

**DOI:** 10.1186/1471-2466-10-46

**Published:** 2010-09-10

**Authors:** Avani C Modi, Alexandra L Quittner, Michael P Boyle

**Affiliations:** 1 Cincinnati Children's Hospital Medical Center, Center for the Promotion of the Treatment Adherence and Self Management, 3333 Burnet Ave. MLC-7039, Cincinnati, OH, 45229, USA; 2 University of Miami, Departments of Psychology & Pediatrics, 5665 Ponce de Leon Blvd Coral Gables, FL 33146, USA; 3 Johns Hopkins University School of Medicine, Johns Hopkins Adult Cystic Fibrosis Program, 1830 E. Monument Street, 5th floor, Baltimore, MD 21205, USA

## Abstract

**Background:**

As more patients with cystic fibrosis (CF) reach adulthood and participate in age-appropriate activities (e.g. employment, dating), disclosure of medical status becomes more important. This study assessed rates of disclosure and its perceived impact on relationships using the Adult Data for Understanding Lifestyle and Transitions (ADULT) online survey.

**Methods:**

Adults with CF participated in the survey via the United States national network of CF Centers. Descriptive and inferential statistics were utilized.

**Results:**

Participants (n = 865) were more likely to disclose to relatives (94%) and close friends (81%) than to dating partners (73%), bosses/supervisors/teachers (51%) or co-workers (39%). Respondents generally reported a neutral/positive effect on relationships following disclosure. Negative effects of disclosure were infrequent, but more likely with dating partners or bosses/supervisors/teachers. Results also indicated that disclosure may be influenced by severity of lung disease and gender, with those having normal/mild lung disease less likely to disclose their diagnosis to both co-workers (p < 0.01) and bosses/supervisors/teachers (p < 0.01), and women being more likely to disclose to close friends (p < 0.0001) and dating partners (p < 0.05) than men.

**Conclusions:**

Most adults with CF disclosed their disease to relatives and close friends. Individuals with severe CF lung disease were more likely to disclose their diagnosis to coworkers and supervisors/teachers. It may be helpful to provide support for disclosure of disease in situations such as employment and dating.

## Background

Due to the early onset and progressive nature of cystic fibrosis (CF), as well as the relatively short life expectancy, CF was previously considered a childhood disorder. However, earlier diagnosis and the development of new treatments over the past two decades have considerably improved the survival of patients with CF. Results from the United States (US) CF Foundation 2008 Patient Registry report indicated that median life expectancy of patients with CF has risen from approximately 25 years in 1985 to over 37 years in 2008[1]. Furthermore, the percentage of patients with CF aged 18 years or older has risen from 30% in 1990 to over 46% in 2008, leading to a greater emphasis on the acquisition of adult roles[1].

Coupled with an improved life expectancy, patients with CF are more likely to seek independence from their families and pursue typical adult activities (e.g. attending college, entering serious relationships and pursuing careers)[2]. During adolescence, individuals want to feel 'normal' and reduce the sense that they are different from their peers. This may lead them to keep disease status a 'secret' or hide visible aspects of the disease (e.g. suppressing cough)[3]. However, in their transition to adulthood, different social contexts and relationships may require disclosure of their CF[4]. For example, concerns about employer-based health insurance may need to be discussed when starting a new job. Similarly, involvement in romantic relationships requires disclosure for long-term planning, including marriage and children. While the area of disclosure has been a focus of research in several other diseases including HIV/AIDS, epilepsy and mental health[5-9], little research has been conducted on disclosure in CF, particularly during emerging adulthood [4,10].

In order to better understand these issues, the Adult Data for Understanding Lifestyle and Transitions (ADULT) survey was conducted. The purpose of the current study was to examine how adults with CF perceive the disclosure process within specific personal and professional relationships, and to better understand the relationship of disease severity and gender to the disclosure process.

## Methods

### Participants

Any adult aged ≥18 years with CF in the US could complete this online survey. There were no additional inclusion/exclusion criteria.

### Procedures

From July 2007 to August 2007, adults with CF were invited via the United States (US) national network of CF Centers to participate in an anonymous, online survey. The survey consisted of five screening and 50 lifestyle questions. Responses to medical (forced expiratory volume in 1 second [FEV_1_], lung transplant and intravenous [IV] treatment during the previous 12 months) and screening questions were required for inclusion in the dataset. The online survey was hosted by GfK Roper (New York, NY, US) and $50 was given to each participant who qualified and completed the survey, with safeguards in place to prevent multiple responses per patient. All authors sent the current protocol to their respective Institutional Review Boards, with each IRB deeming that approval was unnecessary due to the de-identified nature of the anonymous survey. Consent to participate was implicit to adult participants based on completion of the survey.

### Measure

#### Adult Data for Understanding Lifestyle and Transitions (ADULT) survey

The survey consisted of 5 screening and 50 lifestyle questions, encompassing aspects of adult lifestyle and transitions. Major survey categories included questions about education, employment, activities, travel, financial, disability, independent living, social relationships, children, general health, disclosure to others, satisfaction with life, medical information and history, and advice to others with CF. For purpose of the current study, questions regarding demographics (e.g., gender, age), medical information (e.g. FEV_1 _% predicted, time since diagnosis), disclosure, and challenges related to disclosure were utilized. The two questions specific to disease disclosure were: a) "To what extent do the following people know that you have CF: Relatives, Close friends, Dating Partners, Neighbors, Bosses/supervisors/teachers, Co-workers, and Acquaintances?" Response options were as follows: "None of them", "A few of them", "Some of them", "Most of them", "All of them", or N/A; b) "After these people became aware that you have CF, how did it affect your relationship? Relatives, Close friends, Dating Partners, Neighbors, Bosses/supervisors/teachers, Co-workers, and Acquaintances?" Response options were as follows: "Negative", "Mostly negative", "No effect", "Mostly positive", "Positive", or N/A. An additional question related to romantic partners was as follows: "Which of the following challenges related to CF, if any, have you discussed with your partner prior to making this commitment?" Participants responded with a "yes" or "no" to items.

### Statistical analyses

Data were analyzed using descriptive and inferential statistics. Unweighted demographic data are presented to accurately reflect the population completing the survey. All other data were weighted (using standard, iterative sample balancing techniques) by region, age and gender to match national adult CF norms, as provided by GfK Roper from ongoing proprietary research it conducts among patients with CF. This was done to facilitate interpretation of the responses with regard to national CF norms, without potentially confounding sample biases (e.g. disproportionately high number of males versus females compared with national norms). Between-group analyses for disease severity and gender were conducted using Chi-square tests and only patients for whom the question was appropriate were included in analyses (e.g. only those who reported having co-workers were included in the analysis regarding differences in disclosure to coworkers). Disease severity was defined as follows[1]: FEV_1 _% predicted 91-100 = Normal; 71-90 = Mild; 41-70 = Moderate; ≤ 40 = Severe. Effect sizes were calculated from Cramer's phi and then converted to Cohen's d to aid interpretation, with 0.20, 0.50, and 0.80 representing small, medium, and large effects, respectively. Significance was defined as *p *< 0.05.

## Results

### Participant characteristics

In total, 865 completed surveys were included from across the United States (US). This represented approximately 8% of the US adult CF population in the Cystic Fibrosis Foundation Patient Registry[11]. Study participant characteristics are presented in Table 1.

**Table 1:** Study participant characteristics

Variable	Mean (SD)/Percentage
**Age**	*M *= 29.9 ± 9.7 years; Median = 26.5 years; Range = 18-70 years
**Age of diagnosis**	5.8 (± 10.1) years; (76%; n = 658) diagnosed at ≤ 5 years of age
**% Male**	46%
**% Race**	
Caucasian	95%
African American	2%
Hispanic	2%
Other	1%
**% Education Level**^**a**^	
Less than high school	5%
High school graduate	20%
Technical/Professional training	6%
Some college/college student	34%
College graduate	24%
Have/working on graduate degree	11%
**% Marital Status**	
Single (not currently dating)	25%
Single (dating)	15%
Serious relationship	24%
Married	36%
**% Disease Severity**^b^	
Normal (> 90 FEV_1 _% predicted)	16%
Mild (70-90 FEV_1 _% predicted)	24%
Moderate (40 - 69 FEV_1 _% predicted)	29%
Severe (≤ 39 FEV_1 _% predicted)	15%

Note. ^a^Patients between 18-24 may not have the ability to have higher education level based on age. Education level thus represents the highest degree achieved to date. ^b^Disease severity was unknown for 16% of the patients.

### Overall disclosure by relationship group

**Figure 1:**
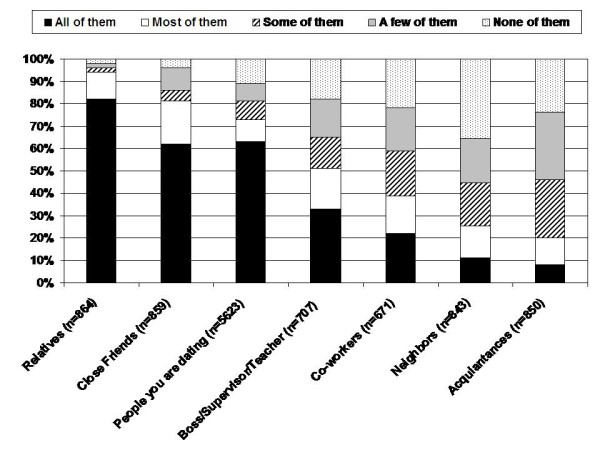
**Degree of disclosure by relationship group**.

When evaluating the 'all' or 'most of' response options, respondents were most likely to disclose to relatives (94%) and close friends (81%; Figure 1). Fewer patients reported disclosure to dating partners (73%), bosses/supervisors/teachers (51%), co-workers (39%), neighbors (25%) and acquaintances (20%).

### Disclosure by lung function

**Figure 2:**
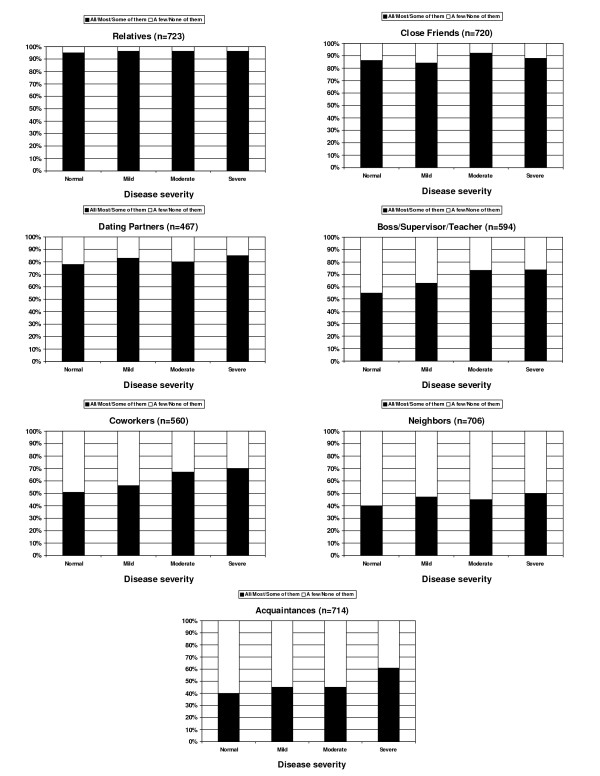
**Degree of disclosure by lung function**. Pulmonary function categories reflect those defined by the CF Foundation[1]; severe = FEV_1 _≤40% predicted; moderate = FEV_1 _41-70% predicted; mild = FEV_1 _71-90% predicted; normal = FEV_1 _> 90% predicted; FEV_1 _= forced expiratory volume in 1 second

Results indicated that while most adults reported that they disclose their CF to relatives and close friends, disclosure to others was influenced by severity of lung disease (Figure 2). Patients with normal and mild disease were less likely to have disclosed (e.g., 'few' or 'none' responses) to their co-workers (49 and 44%, respectively) than those with moderate and severe disease (33 and 30%, respectively; χ^2 ^(3, 561) = 11.87, *p < .01; effect size Cohen's d = 0.29*). Similarly, patients with normal and mild disease were less likely to have disclosed (e.g., 'few' or 'none' responses) to their bosses/supervisors/teachers (45 and 37%, respectively) than those with moderate and severe disease (27% and 27%, respectively; χ^2 ^(3, 593) = 14.68, *p < .01; effect size Cohen's d = 0.32*). Conversely, a significantly greater proportion of patients with severe disease had disclosed to at least some acquaintances (60%) compared with patients with moderate (45%), mild (45%), and normal disease (40%) (χ^2 ^(3, 714) = 12.70, *p < .01; effect size Cohen's d = 0.27*). No significant differences in disclosure by disease severity were found for friends, relatives, dating partners or neighbors.

### Perceived effects of disclosure on relationships

**Figure 3:**
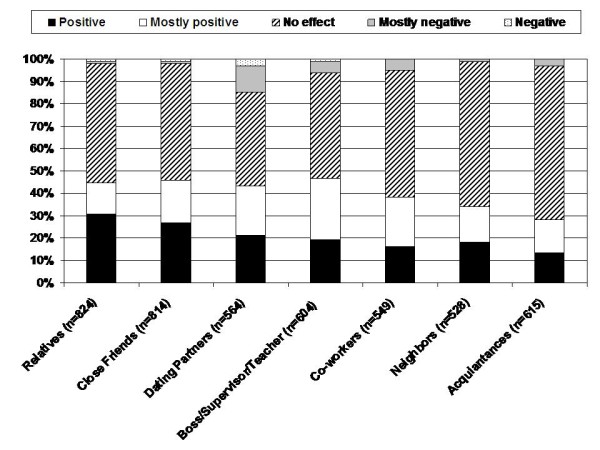
**Perceived effect of disclosure on relationships**. Filtered for patients with identified relationships within each group for which CF was disclosed

Patients reported that disclosure of their disease to others generally had a neutral or positive effect on their relationships (Figure 3). Relationships with relatives or close friends were most likely to be positively affected by disclosure (45 and 46%, respectively). Negative effects of disclosure on relationships were reported infrequently; however, a negative effect was most likely to be reported for dating partners or bosses/supervisors/teachers (15% and 6%, respectively).

### Gender differences in disclosure and challenges discussed with partner following disclosure

There were no significant differences by gender in disclosure to relatives, neighbors, acquaintances, bosses/supervisors/teachers, and coworkers. However females were more likely to disclose (e.g. all/most of the time) to both close friends (females 87% vs males 75%, χ^2 ^(1, 858) = 17.1, p < 0.0001; *effect size Cohen's d *= 0.29) and dating partners (females 52% vs males 43%, χ^2 ^(1, 563) = 4.91, p < 0.05; *effect size Cohen's d *= 0.19). To better understand disclosure while dating, patients with serious partners (e.g. married, engaged or planning to live together; n = 548) were asked to identify the most important issues they had discussed prior to making a commitment. The most commonly discussed challenge was the daily, time-consuming requirement for CF treatments (Table 2). Men were significantly more likely than women to discuss fertility issues (86% versus 77%, respectively; χ^2 ^(1, 549) = 6.78, *p < .01; effect size Cohen's d = 0.22*), whereas women were more likely to discuss healthcare cost/benefits (85% versus 75%, respectively; χ^2 ^(1, 549) = 8.63, *p < .01; effect size Cohen's d = 0.25*), financial issues (83% versus 72%, respectively; χ^2 ^(1, 549) = 9.34, *p < .01; effect size Cohen's d = 0.26*) and birth control methods (83% versus 65%, respectively; χ^2 ^(1, 548) = 23.40, *p < .001; effect size Cohen's d = 0.42*). No other significant gender differences were noted for other common challenges, such as performing treatments, infection risk, or risk of CF in future children.

**Table 2:** Challenges related to cystic fibrosis discussed with partners prior to making a serious commitment

Discussion topic	Frequency of endorsement, %
Daily CF therapy	88

Possible time in hospital	86

Risk of CF in future children	82

Fertility issues	81

Healthcare costs/benefits	80

Financial issues	78

Infection risk	74

Birth control methods	74

Note. Filtered for patients who were either married, getting married in the near future or planning to move in with a partner (n = 548).

## Discussion

While the challenges of disclosure of illness have been a focus of research in HIV/AIDS, epilepsy and mental health, minimal research has been conducted on disclosure in CF [4-10]. Studies in other diseases have suggested that two of the strongest influences on the decision to disclose are concern about social stigma and increasing health needs driven by disease severity [5,7,8]. Increasing survival of patients with CF and thus, greater involvement of adults with CF in age-appropriate activities have made disclosure an increasingly important topic in CF. The ADULT survey offered insight into the activity and disclosure practices of a large, representative sample of the US adult CF population.

Overall, results from the ADULT survey suggested that most adults with CF are pursuing typical age-appropriate activities, such as employment, independent living, romantic relationships and marriage [2]. Two particularly interesting aspects of this study were the perceived effects of CF disclosure on relationships and how these vary by type of relationship, disease severity, and gender. The decision to disclose CF was shown to be influenced by these factors, which are likely linked to perceptions of the risks and benefits of disclosure [3,4,12,13].

Specifically, patients were more likely to disclose their condition to relatives and close friends than dating partners, supervisors/employers/teachers or co-workers. Among relatives and close friends, it is likely that some level of personal intimacy has been established, making it easier to disclose one's medical condition. Relatives and close friends also provide significant levels of support for adults with CF in terms of disease management, clinic visits and hospitalization[14]. Thus, it is essential to disclose one's condition to family members and close friends, who may be needed in emergency situations.

Disclosure to employers or potential romantic partners appear, however, be more complicated. These situations are directly related to the personal goals of the adult with CF and, therefore, the perceived risk of a negative reaction to disclosure is heightened. For example, when pursuing a romantic relationship, patients may be concerned about fertility issues[15], whereas in the workplace, they may be concerned about their own perceived limitations in relation to healthy colleagues (e.g., needing time off for clinic visits or hospitalizations)[4]. In both situations, patients may choose to conceal rather than disclose their condition because of the potential risks of rejection. Although disclosure of CF is not legally required for employment, there may be situations in which it is beneficial (e.g. letting employers know why you are absent from work). Therefore, we encourage CF teams to have a dialogue with their patients about disclosure and the risk/benefit ratio of disclosure in these contexts.

Severity of lung disease also influenced respondents' approach to disclosure. More patients in the moderate and severe disease groups disclosed to bosses/supervisors/teachers, compared to patients with normal and mild disease. These results are consistent with prior literature, suggesting that disclosure is more likely for those with severe disease because the condition is more difficult to conceal (e.g. more coughing or fatigue)[4]. Thus, Troster's observation that disclosure is related to perceived risk of detection is supported by data from this large cohort of adults with CF[12]. Specifically, patients with mild disease may consider their symptoms less obvious and their bodily appearance/function more 'normal' in comparison to those with more severe disease and, therefore, feel less need to risk disclosure of their condition and its potential consequences.

Gender differences in disclosure were also found. Specifically, females were more likely to disclose to close friends and dating partners than males. This is consistent with studies showing that females with chronic conditions are more likely to disclose their disease status than males [5,9]. Women were also more likely to discuss issues related to healthcare, finances, and birth control with their partners than men. One possible reason is that disclosure in this context may elicit social support from close friends and dating partners, which is beneficial for their disease management.

Disclosure to neighbors and acquaintances was generally the lowest across types of relationships and disease severity groups. However, disclosure to at least some individuals within these groups was greater in those with more severe disease. Patients with fewer symptoms and a more 'healthy' appearance may feel that disclosure in these situations is more bothersome than helpful[4]. Specifically, the public's general knowledge of CF is relatively low compared with other chronic illnesses and, therefore, rather than explain CF in transient relationships, it may be easier to relate milder symptoms to a chest infection or a better recognized disease, such as asthma. However, as symptoms and bodily appearance worsens, becoming potentially more noticeable in casual encounters, some patients preferred to disclose their diagnosis rather than being perceived as having a stigmatizing disease, such as HIV/AIDS.

Our finding that disclosure is associated with a perceived neutral or positive effect on most relationships supports a previous study indicating that lack of disclosure can negatively impact peer evaluations[13]. Specifically, without an awareness of an individual's diagnosis of CF, peers may attribute symptoms or social absences to other problems, which can lead to negative attributions and appraisals. For example, being shorter and thinner than peers, or taking enzymes with food, may be misunderstood by observers who are unaware of the person's condition. Thus 'preventative disclosure' [10], as it is termed, may have a positive influence on relationships by increasing awareness and reducing misperceptions. By definition, however, preventative disclosure requires an analysis of the potential positive and negative effects of disclosure versus continued concealment. Given that some perceived negative effects of disclosure were reported for supervisors/employers/teachers or romantic partners, it may be important for providers to discuss disclosure in these social contexts.

## Conclusions

The majority of adults with CF are now pursuing typical, age-appropriate adult activities that may require disclosure of their medical condition. The results of the ADULT survey suggested that, although most adults with CF choose to disclose their disease to relatives and close friends, those with mild lung disease are less likely to do so with supervisors/employers/teachers and co-workers. On average, adults reported that disclosure was associated with a perceived positive or neutral effect, with a few negative responses occurring with dating partners and employers. Healthcare providers may want to counsel adult patients with CF on strategies for disclosure of their condition in these more high-risk situations.

## Competing interests

Avani Modi, PhD, has served as a consultant for Novartis Pharmaceuticals Corporation.

Alexander L. Quittner, PhD, has served as a consultant for Novartis Pharmaceuticals Corporation, PTC Therapeutics, Gilead Sciences, Transave and Vertex; received a research grant from Novartis Pharmaceuticals Corporation, for 2008-2012; has been a speaker for Novartis Pharmaceuticals; is a member of the North American Advisory Group, Genentech, Inc.

Michael P. Boyle, MD: has served as a consultant for Novartis Pharmaceuticals Corporation.

## Authors' contributions

All authors (AM, AQ, MB) contributed to the study design, development of the ADULT survey, drafting of the manuscript, and have given final approval of the version submitted for publication/published.

## Pre-publication history

The pre-publication history for this paper can be accessed here:

http://www.biomedcentral.com/1471-2466/10/46/prepub
